# Does adding coaching calls into an online lifestyle intervention for cancer survivors make it more acceptable and feasible than a self-guided version?

**DOI:** 10.1007/s00520-025-10263-9

**Published:** 2026-01-22

**Authors:** Nicola Freeman, Morgan Leske, Bogda Koczwara, Julia Morris, Anthony Daly, Lisa Beatty

**Affiliations:** 1 Flinders University Institute of Mental Health and Wellbeing, College of Education, Psychology and Social Work, Flinders University, Adelaide, SA Australia; 2 Flinders Health and Medical Research Institute, College of Medicine and Public Health, Flinders University, Adelaide, SA Australia; 3 Department of Medical Oncology, Southern Adelaide Local Health Network, Adelaide, SA Australia; 4 Cancer Council SA, Eastwood, SA Australia

**Keywords:** Cancer survivors, Digital intervention, Guidance, Coaching calls, Qualitative feedback

## Abstract

**Objectives:**

While online interventions increase access to support, self-guided online programs have lower engagement and (potentially) efficacy than guided programs. However, the acceptability of guided programs to cancer survivors is less established. This study qualitatively explored whether the addition of guidance via two brief coaching calls to the Healthy Living after Cancer (HLaC) *Online* program, an online lifestyle intervention, was feasible and more acceptable than the self-guided version.

**Methods:**

Participants were adult Australian cancer survivors randomized to receive either HLaC *Online* + coaching (*n* = 25) or HLaC *Online* (*n* = 27) as part of a larger clinical trial. Semi-structured telephone interviews were conducted (*n* = 21), and transcripts were analyzed via framework analysis, using deductive analysis of four a priori feasibility domains (adaption, acceptability, demand, practicality) and inductive analysis to generate novel subthemes. Recruitment ceased when content saturation was achieved.

**Results:**

Feedback suggested guidance was a positive *adaption*, with benefits including high *acceptability*, *practicality* through program understanding, and maintenance of behavior change. Control participants reported high *demand* for coaching calls and lower acceptability.

**Conclusions:**

Feedback suggested that Australian cancer survivors found coaching calls acceptable and feasible, more so than the self-guided program. HLaC *Online* + coaching supported Australian cancer survivors to interact with the program more effectively than without coaching and achieve reported benefits in both physical and psychosocial health, suggesting better survivorship outcomes. Future research should implement the addition of coaching calls at a larger scale, to establish whether calls impact efficacy and engagement.

## Introduction

There are more than 1 million cancer survivors in Australia [1]. The number of cancer survivors is expected to increase, not only in Australia but worldwide, due to advancements in technology, screening, and treatments [2, 3]. Yet, cancer survivors still face many lifestyle and personal challenges in life after cancer, including chronic fatigue, pain, mental health challenges, cancer recurrence and mortality, and/or co-morbid conditions [2–5]. Maintaining a healthy lifestyle after cancer, via regular physical activity and a healthy diet, can help reduce these adverse effects and enhance quality of life (QoL) [6, 7]. However, improving health behaviors can be difficult to enact and sustain due to factors such as low motivation and post-treatment fatigue [8]. In Australia, cancer survivors have low long-term adherence to health guidelines, such as physical activity and diet recommendations [9], as well as poorer physical health compared to people who have not received a cancer diagnosis [10].

Notably, when considering healthy lifestyle guidelines, cancer survivors define a healthy lifestyle as incorporating both physical and mental health strategies [11]. This is unsurprising, given a cancer diagnosis negatively impacts mental health outcomes, such as elevating distress [12] and reducing QoL [13], with survivors frequently reporting unmet needs in these domains [14]. Furthermore, depression and anxiety can increase the risk of cancer mortality in multiple cancer types by more than 20% [15], while high QoL can act as a protective factor against recurrence and mortality [6]. Therefore, both physical health and psychosocial support are critical to address in any healthy lifestyle program throughout cancer survivorship to optimize survivorship outcomes [14].

While traditional face-to-face interventions are demonstrably efficacious, they are not regularly implemented throughout cancer survivorship due to cost and resource burden [16]. Further, face-to-face delivery is not accessible to all populations, including those living in rural and remote areas with reduced access to healthcare [17]. To address the need for accessible interventions that cover both physical and psychosocial health factors, the Healthy Living after Cancer (HLaC) *Online* program was developed [18]. This program was an end-user co-designed adaption of the original telephone-delivered intervention [19]. HLaC *Online* is a 12-week web-based program, comprised of nine healthy living modules (My Goals, Finding the New Normal, Physical Activity, Healthy Eating, Mental Health, Fatigue Management, Maintaining a Healthy Weight, Peer Support, Staying on Track) and five progress trackers (exercise tracker, meal tracker, weight tracker, thought record, and mood and fatigue monitor) that post-treatment cancer survivors can access to help achieve physical, dietary, and psychosocial goals. Despite being co-designed using end-user feedback, the initial feasibility study for HLaC *Online* demonstrated low user uptake and engagement in the program [20]. Qualitative feedback suggested this may be a consequence of the self-guided nature of the program and that users might benefit from guidance calls to assist with program navigation, setting up health behavior goals, and accountability [20]. This aligns with recent meta-analytic evidence that guided digital interventions have higher engagement, as well as higher efficacy in the domains of anxiety, fatigue, and distress [21].

Our team has recently conducted a feasibility trial of adding two coaching calls to HLaC *Online* (HLaC *Online* + coaching) compared to the unguided version [22]. A stakeholder meeting with Cancer Council SA, an Australian not-for-profit cancer support organization, was held, where the addition of two phone calls was suggested based on a randomized control trial (RCT) by Evans et al. [23], and similarly, Cancer Council concluded two calls would be the feasible dose for potential post-study implementation of HLaC *Online* + coaching [24]. Although quantitative feasibility and efficacy results from the trial are yet to be published, preliminary findings of the RCT suggest positive long-term lifestyle outcomes for cancer survivors accessing HLaC *Online* + coaching [22]. Yet, it is unknown whether adding this guidance is experientially acceptable and beneficial to cancer survivors. Qualitative data provides opportunities to investigate more than just *whether* an intervention is efficacious, but *why*, and obtains important feedback from end-users. As acceptability can predict higher digital intervention engagement and effectiveness [25, 26], exploring the acceptability of HLaC *Online* + coaching is essential to help understand the intervention’s impact. Thus, the purpose of this study was to qualitatively explore whether the addition of guidance via two brief coaching calls to HLaC *Online* resulted in higher feasibility and acceptability than the self-guided version.

## Method

The full protocol outlining the methods, measures, and planned analyses for the feasibility RCT has been prospectively registered (ANZCTR, ACTRN12622001111763). In the following, methods relevant to the qualitative analysis of HLaC *Online* + coaching’s feasibility and acceptability are summarized.

### Participants

Participants in this sub-study were Australian cancer survivors over 18 years old who had successfully enrolled in the HLaC *Online* + coaching feasibility RCT, recruited between 23/08/22 and 17/01/23. With ethics approval (HREC, 2106), participants from both the guided and unguided conditions were invited to participate in a telephone interview, with interviews following the completion of the 12-week intervention period. Potential participants were contacted via telephone, SMS, or email a maximum of three times and were recruited until content saturation of themes occurred.

### Intervention conditions

Both intervention (HLaC *Online* + coaching) and active control (HLaC *Online*) participants received the 12-week HLaC *Online* program. The program included access to nine modules and five trackers as previously specified. During the program, participants received weekly SMS reminders to log into the program and two additional email reminders if they had not logged on in 1 or 2 weeks. However, only intervention participants received two additional telephone coaching calls, the first in week 1 and the second in week 4. The week 1 call orientated participants to the website, discussed healthy lifestyle aims, and set SMART (specific, measurable, achievable, relevant, time-bound) goals with signposting to relevant modules. The week 4 call acted as a check-in, where participants discussed their progress, barriers encountered, and how to overcome them, and addressed any questions arising about the program. All coaching calls were facilitated by one coach, a doctoral level provisional psychologist with cancer care experience, and followed a script that had been discussed and reviewed during the Cancer Council SA stakeholder meeting [24].

### Procedure

Consenting participants were provided with a topic guide (Table 1) prior to the interview. During the interview, they were asked about their satisfaction with, and the usability of, the program and coaching calls (if relevant). Interviews were conducted by two researchers (NF, DK), psychology students undertaking their honors or work experience placement (respectively) under the supervision of senior authors (ML, LB). Interviews were audio-recorded, transcribed verbatim, and coded. Saturation was determined through an iterative process after each interview; after completing two consecutive interviews with no new data emerging, content saturation was deemed to have occurred. Due to the high information power of the study population [27], saturation was able to be achieved with fewer interviews [28].

**Table 1:** Topic Guide for Telephone Interviews

Section		Questions and Prompts
Section 1: Accessing the program	*This section asks questions about how you accessed Healthy Living after Cancer Online.*	· Did you receive the program as self-guided or with the telephone coaching calls? · What device did you usually use the program on?
Section 2: Satisfaction with program overall	*This section asks questions about how you used Healthy Living after Cancer Online and to provide your feedback on the program overall.*	· How did you find the program overall? · Is there anything you would change to the look of the program? · How did you find navigating the website? · What aspects of Healthy Living after Cancer Online were the most useful to you? · What aspects of Healthy Living after Cancer Online were not useful? · Are there any topics that are missing which you would like addressed in this program? · How did you find the email and text reminders? · [If you received the telephone coaching calls] How did you find the telephone coaching calls?
Section 3: Trackers	*This section asks you to provide your feedback on each of the trackers included in the Healthy Living after Cancer Online program.* *When providing your feedback on each of the trackers, you may consider the following questions:* - *How much did use this tracker? * - *How helpful did you find this tracker? * - *Is there anything you would change about this tracker? * *If you did not use one or more of the trackers, that is okay. You may also provide some feedback about why you chose not to use the tracker. *	· Exercise tracker · Meal tracker · Weight tracker · Thought record · Mood and fatigue monitor
Section 4: Intervention modules	*This section asks you to provide your feedback on each of the modules included in the Healthy Living after Cancer Online program. When providing feedback on each of the modules, you may consider the following questions:* - *Overall, what did you think of this section? * - *How relevant was the information in this section? * - *How helpful was the information is this section? * - *Was the information easy to understand? * - *What did you think of the activities included in this section? * - *Is there anything you would change about this module? * *If you did not use the module, that is okay. You may also consider providing feedback about why you chose not to use the module. *	· Finding the new normal · My goals · Physical activity · Healthy eating · Mental health · Fatigue management · Maintaining a healthy weight · Staying on track · Peer support · Any other feedback?

### Statistical analysis

Participant demographic and clinical characteristics were summarized using descriptive statistics. Qualitative data were coded using NVivo R1 software [29]. Two transcripts were coded independently by two researchers (NF, ML) and compared to confirm consistency of themes and establish a coding framework. The remaining 19 transcripts were coded independently by one researcher (NF). Codes were collated into emergent themes and subthemes using framework analysis [30], a type of thematic analysis where an existing framework is applied to guide and categorize responses. It enables both deductive (top down, coding themes to a priori established categories) and inductive (bottom up, identifying new emergent themes that do not fit existing categories) analyses. As the HLaC *Online* RCT was a feasibility study, Bowen and colleagues’ [31] feasibility framework was applied to analyses, with four key a priori domains of focus: (i) *adaption*; (ii) *acceptability*; (iii) *demand*; and (iv) *practicality*.

Data analysis was guided using the six stages of thematic analysis outlined by Braun and Clarke [32]: Stage 1 (familiarization with the data), Stage 2 (initial coding of raw data into specific groups), Stage 3 (categorizing these codes/groups into main themes), Stage 4 (reviewing themes and checking that they were representative of the data set for both conditions), Stage 5 (finalizing and defining themes), and Stage 6 (writing the report itself). As recommended by Braun and Clarke [33], reflexivity was monitored throughout the analytic process via conversations with researchers experienced in qualitative data analysis, active notetaking, and reflection to ensure no personal biases shaped the research. Results were summarized by intervention/control group assignment.

## Results

Of the 52 participants in the parent RCT, 21 completed qualitative interviews (11 intervention, 10 control). Table 2 presents participant demographic and clinical characteristics. Participants were aged between 35 and 73 years, with 15 residing in urban communities and six in rural areas. Two-thirds of participants were highly engaged in the website and had completed five or more modules during the intervention period. Three participants (1 intervention, 2 control) did not complete any modules. Average interview time was 27.38 minutes, with the length of interviews ranging from 12 to 42 minutes.

**Table 2:** Participant demographic and clinical characteristics

Characteristic	HLaC Online + coaching (*n* = 11)	HLaC Online (*n* =10)	Overall (*n* = 21)
Age	*M* (*SD*)	*M* (*SD*)	*M* (*SD*)
	57.51 (9.61)	58.40 (13.18)	57.94 (10.13)
Age at diagnosis	51.33 (9.29)	51.50 (13.45)	51.42 (11.35)
Time since diagnosis (years)	4.22 (4.10)	6.43 (8.69)	5.39 (6.82)
	*n *(%)	*n *(%)	*n *(%)
Relationship status			
Married/Defacto	7 (63.60)	9 (90.00)	16 (76.20)
Other^a^	4 (36.40)	1 (10.00)	5 (23.80)
Educational achievement			
Secondary school	-	-	-
TAFE	1 (9.10)	2 (20.00)	3 (14.30)
Tertiary	10 (90.90)	8 (80.00)	18 (85.70)
Country of Birth			
Australia	8 (72.70)	5 (50.00)	13 (61.90)
Other^b^	3 (27.30)	5 (50.00)	8 (38.10)
Cultural Background			
Australian	9 (81.81)	8 (80.00)	13 (61.90)
English	3 (27.27)	1 (10.00)	4 (19.00)
Other^c^	3 (27.27)	-	3 (14.30)
Location			
Urban	7 (63.60)	8 (53.30)	15 (71.40)
Rural	4 (36.40)	2 (20.00)	6 (28.60)
Cancer type			
Breast	10 (90.90)	9 (90.00)	19 (90.50)
Lymphoma	-	1 (10.00)	1 (4.75)
Thyroid	1 (9.10)	-	1 (4.75)
Completed Treatment			
Yes	11 (100)	8 (80.0)	19 (90.50)
No/Unsure^d^	-	2 (20.00)	2 (9.50)
Treatment received^e^			
Surgery	11 (100)	9 (90.00)	20 (95.20)
Chemotherapy	10 (90.90)	8 (80.00)	18 (85.70)
Radiotherapy	8 (72.70)	6 (60.00)	14 (66.70)
Immunotherapy	1 (9.10)	4 (40.00)	5 (23.80)
Hormonal therapy	9 (81.80)	5 (50.00)	14 (66.70)

^a^Divorced (*n* = 4) and Missing (*n* = 1)

- indicates no participants in this group.

^b^United Kingdom (*n* = 5), Canada (*n* = 1), New Zealand (*n* = 1), and South Korea (*n* = 1).

^c^ Korean (*n* = 1), Irish (*n* = 1), and Scottish (*n* = 1).

^d^ All participants selecting ‘No’ or ‘Unsure’ were currently on hormonal therapy

^e^ Multiple responses allowed.

### Qualitative findings

**Fig. 1:**
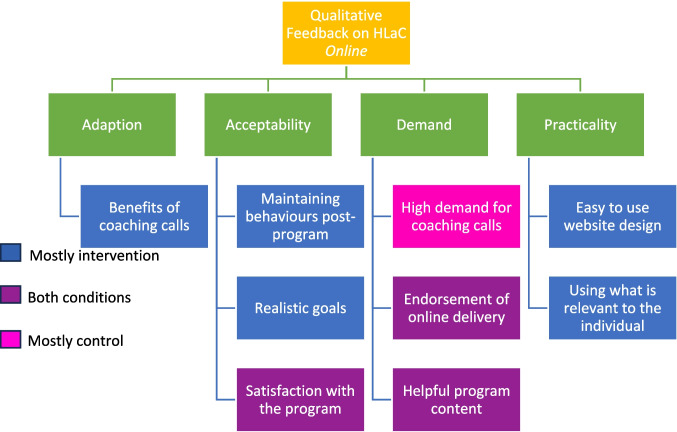
Identified themes and subthemes from HLaC online feedback

Figure 1 displays the final thematic map, where primary themes followed Bowen and colleagues’ [31] feasibility framework: adaption, acceptability, demand, and practicality. There were up to three subthemes for each, which were further color coded to display which group provided the dominant feedback.

#### Adaption

Adaption is related to how an existing program performed when changes were made, this being the addition of two telephone coaching calls. This subtheme was only raised by intervention participants, with one subtheme, *benefits of coaching calls.*

##### Benefits of coaching calls

Intervention participants universally commented on the calls’ benefits, describing calls as “helpful,” “valuable,” and even “critical.” Several participants described the benefits of having a personal connection through the calls:

Intervention participant, female, 63 years: I think definitely that the personal side of having the calls, you weren’t just client number 479 going through a computer program, you were, you actually became a person. And I think bouncing off someone else helped clarify things. Yeah, I found those very, most valuable.

The importance of contact for motivation was also discussed, with one participant describing that the calls helped in “setting yourself up for success” by having someone “that holds you a little bit to account.” The calls also encouraged participants to continue using the program, with one participant stating they “probably would have given up if I didn’t have that call.” Thus, the calls appeared to re-commit participants:

Intervention participant, female, 48 years: I think having the call really refocused me and encouraged me to keep going, and it also really emphasised that the program is called Healthy Living with – after – Cancer, that idea of, you’ve got to still live. If you have a little set back it’s okay, just get up and keep going in the morning. That call, it was absolutely critical for me.

#### Acceptability

Acceptability is related to the reaction of participants to the program regarding suitability, satisfaction, and appropriateness, as well as positive or negative impacts. This theme was raised by both conditions. Subthemes included *maintaining* *behaviors* *post-program*, *realistic goals*, and *satisfaction with the program*.

##### Maintaining behaviors post-program

While the ability to maintain behaviors initiated during the HLaC *Online* program in post-program life was mentioned by both groups, this was predominantly discussed by intervention participants. Several participants attributed this maintenance to practical skills learned during the program, ensuring they were “armed with strategies” for success.

##### Realistic goals

While being able to create realistic SMART goals was mentioned by both groups, it was raised substantially more often by intervention participants. One intervention participant described that “the most useful [aspect] for affecting positive change within myself was definitely the goal setting” and that the module itself paired with the coaching calls “really helped not just set goals but set realistic goals.” Thus, coaching calls appeared to be particularly valuable in breaking down goals:

Intervention participant, female, 64 years: I thought that was a really good part of the program … that you had [coach’s name] going through with you … giving you a really good understanding of what a goal is, how you can set it, and then … sort of going through the little steps that you can achieve it. Because sometimes, it’s all very well reading things, but if you haven’t got a practical, a real practical example with another person on the line that you can actually say, “well hold on, have I got that right?”.

For control participants, feedback indicated that a call to help orientate themselves could have helped overcome any initial problems and to set more realistic SMART goals.

##### Satisfaction with the program

Satisfaction with the program was raised equally by intervention and control participants, describing the program as “helpful, easy to understand” and “very informative.” One control participant highlighted that “everyone should have access to… support, and that’s what your platform does, is give everyone access.” Intervention participants further commented on their satisfaction with the calls, with one participant stating that they “thought that was a really, really good aspect of the program.”

#### Demand

Demand is related to how much the program was used, the demand for the program, and participant interest. This theme was discussed by both conditions. Subthemes included *endorsement of* *online delivery*, *high* *demand for coaching calls*, and *helpful program content*.

##### Endorsement of online delivery

Most intervention and control participants were in favor of the online modality of HLaC and equally discussed the demand for such a program online due to its accessibility. Participants reported enjoying that they “could go back to it anytime” and “that it was self-paced to fit in with commitments.” A rural participant specifically commented on the benefits for remote survivors due to COVID-19:

Intervention participant, female, 64 years: and also, because of COVID… I live in a regional area but some people live in a really remote area and they wouldn’t be able to access these things [in person]. So online’s great.

However, one intervention and two control participants (all aged above 50 years) commented that the online format, even with coaching calls, was not their preference. The intervention participant expressed that “having to type in all those letters in on a… little keyboard was just not my cup of tea.” A control participant described that as they worked online, they were “sick of devices.”

##### High demand for coaching calls

A key function of program acceptability and feasibility for intervention participants was coaching calls, and for control, there was a high demand for coaching throughout the program. As participants could not be blinded in the RCT, several control participants described that they “had [their] hopes up on talking to someone.” One control participant emphasized the need for human connection, explaining that they would have been “more diligent and committed” to the program if they received calls.

##### Helpful program content

This subtheme was raised equally by intervention and control participants. Feedback was in favor of the module layout, which allowed participants to use the parts of the program most relevant to them. The most frequently discussed module for both conditions was “Mental Health,” where participants emphasized its importance as “the survivorship journey has been… as much about mental health as it’s been for… physical health.” Another participant pointed out the lack of support for mental health post-treatment, and because of that, including the “Mental Health” module in the program was extremely “valuable”:

Control participant, female, 35 years: That was a really difficult thing to find the resources for, and to get my head around, was how to mentally live. Everyone tells you what you have to do physically and you know, to look after your exercise and… look at your diet and all those sorts of things. But no – there’s just- you feel like you’re supposed to be grateful but actually you’re just utterly traumatised. And yeah, it’s such a difficult thing.

#### Practicality

Practicality related to resources, time, and commitment needed to carry out the program, and the effect on participants was mentioned by both conditions. Subthemes included *easy-to-use* *website design* and *using what is relevant to the individual*.

##### Easy-to-use website design

Feedback on the ease of navigating the website was positive overall, yet intervention participants found navigation easier than control. Several intervention participants described the program as “easy to navigate” and “it was well set out.” While a few control participants also complimented the “very easy” navigation, the intensity with which this was brought up was lower.

##### Using what is relevant to the individual

All intervention participants commented on the practicality of the program due to the module approach, compared to only half of the control participants. General feedback was that “people can dip into it and take what they need,” “do the bits that were relevant to them,” and “that you could do it at your own pace.”

## Discussion

This study qualitatively explored whether the addition of two brief coaching calls to HLaC *Online* resulted in higher perceived feasibility and acceptability than the self-guided version from the perspective of end-users. Qualitative feedback supports the recent meta-analytic findings [21], showing that intervention participants emphasized the benefits and acceptability of coaching calls in setting realistic goals, maintaining behavior change, navigating the program, and feeling more supported, accountable, and motivated. In comparison, self-guided participants reported wanting guidance throughout the program.

Participant feedback indicated that including coaching calls in HLaC *Online* was acceptable and feasible, leading to higher endorsement than the self-guided version. Specific benefits of the coaching calls were the ability to ask questions throughout the program, engage with a facilitator, and increase accountability, these being key factors for continued engagement with the program and setting realistic goals. This is consistent with user feedback from the initial HLaC *Online* feasibility study, which emphasized that users would benefit from calls to increase accountability and help set goals [20]. Feedback is also consistent with meta-analytic conclusions that guidance increased engagement in digital interventions [21] and previous suggestions that a lack of contact and feedback reduce adherence/engagement in digital interventions [34]. Indeed, research highlights that key influences on digital intervention engagement in patient, public, and cancer populations are motivation, personal tailoring and support [35], and increased social support which can be achieved through phone calls [4]. Higher engagement levels relate to increased digital behavioral intervention effectiveness [36], thus participant feedback that coaching calls promoted higher user engagement suggests that HLaC *Online* + coaching could be more effective than the self-guided version. Notably, there is still large heterogeneity in the literature surrounding cancer survivors’ engagement in digital interventions; hence, further high-quality research is needed [37].

A key finding from the present study was that intervention participants reported being able to maintain health behaviors post-program more frequently than the control. This indicates that a benefit of the coaching calls was not only facilitating the initiation of behavior change but also supporting long-term maintenance. A meta-analysis by Leung and colleagues [38] indicated that guided digital mental health interventions produce more effective outcomes than unguided, suggesting that guidance may contribute to better or more enduring effects. The mechanisms through which the coaching calls, combined with the online program, work appear to be via providing opportunities for ongoing troubleshooting, skill consolidation, and maintaining motivation. Whether this translates to more sustained quantitative effects of HLaC *Online* + coaching will be important for future studies to explore.

Intervention participants received just two brief coaching calls, demonstrating that guidance does not need to be exhaustive to be impactful. While brief, these coaching calls provided more guidance than basic technical support, but less guidance than therapy; for example, the coach actively worked with participants to create realistic goals for change. It is noteworthy that intervention participants were satisfied with the number, frequency, and duration of calls provided and did not mention wanting more, while control participants simply discussed wanting contact without specifying how much. This suggests that the dose provided was perceived as appropriate and feasible by end-users. From a sustainability point of view, this dose aligns with Cancer Council SA’s infrastructure, which stated the dose of two calls would be a realistic potential post-study implementation [24]. This contrasts with the original telephone-delivered HLaC program that could not be maintained due to resource heaviness [3]. It is important to note that the optimal dose for guidance has not yet been established [39]; however, the small number and duration of calls are supported by previous studies experiencing implementation challenges, such as calls being time-consuming [3, 40] and participants not answering calls [41]. Thus, additional coaching calls may be unrealistic and unnecessary to implement to achieve adequate user acceptability and satisfaction. Future research could also explore the use of artificial intelligence (AI). Preliminary research using AI coaching suggests this may be a feasible approach; both stand-alone or combined with human support, AI shows initial positive impacts on engagement, accountability, and intervention outcomes [42, 43]. While this is a newly emerging field [42], it could be an avenue to further increase accessibility of support to cancer survivors by reducing resource burden and reliance on training or availability of human coaches.

This study had a number of strengths, including the rigorous approach to analysis and the appropriate qualitative sample obtained with high information power. Drawing on participants from both unguided and guided versions of the program enabled a nuanced comparison of the acceptability, benefits, and impact of coaching vs the digital program itself. This analysis extends quantitative findings that demonstrate *whether* guidance increases engagement, with *why* and *how* intervention participants benefited from the program more than control participants. Specifically, *why* participants benefited included factors such as motivation and accountability, which is in line with the theoretical Model of Supportive Accountability [44]. This model explains how human support increases the effectiveness of online interventions, providing a reference for factors that may benefit digital programs. Hence, findings extend our understanding and are consistent with previous models of the mechanisms that increase the effectiveness of accessible interventions, as well as providing feedback that can help inform future iterations of HLaC *Online*. Furthermore, having only one coach conduct calls increased the consistency of coaching approach and delivery. In line with this, evidence suggests that having a coach be a healthcare professional in the area of focus, in this case, cancer care, has the highest rates of improvement for cancer survivors [45]. It should be noted, though, that one coach does limit generalization, as it is unknown how successful training other coaches to facilitate calls would be; hence, this needs to be further explored.

However, there were some notable limitations. Qualitative interviews were conducted several weeks after participants completed HLaC *Online* in either format, and some participants had stopped using the program earlier than the specified 12-week period. This resulted in a long interval between program use and interviews, which limited the feedback provided by some participants due to their inability to recall the program in detail. Furthermore, interview participants were likely to be high engagers of the intervention and had completed over half of the available modules. Consequently, it is unclear whether these findings are generalizable to participants who engage minimally with online interventions. Finally, coders were not blinded to the condition of participants, leading to potential bias in the form of experimenter expectancies. Yet, reflexivity was used as part of the qualitative analysis process, hence helping account for this bias.

## Conclusion

In conclusion, HLaC *Online* + coaching was acceptable and feasible, more so than the self-guided version. The qualitative feedback from this study strongly supported the inclusion of two coaching calls as a means of maintaining post-program benefits and maximizing survivors’ lifestyle outcomes, confirming and extending the recent meta-analysis findings on the role of guidance in facilitating engagement and outcomes [21]. More broadly, feedback emphasized that HLaC *Online* + coaching increased accountability and a sense of support compared to HLaC *Online*; however, the generalizability of the study is limited due to only one coach and engagement factors. Future research should investigate the routine community implementation of the HLaC *Online* + coaching program at a larger scale, while continuing to obtain end-user feedback to maximize intervention effectiveness. Future program iterations could also explore implementing AI coaching to investigate if acceptability is replicable and resource-burden can be further minimized.

## Data Availability

The datasets analysed in the present study are available from the corresponding author on reasonable request.

## References

[1] Australian Institute of Health and Welfare (December 1, 2021) *Cancer in Australia 2021*, *Summary*. https://www.aihw.gov.au/reports/cancer/cancer-in-australia-2021/summary

[2] Stanton AL, Rowland JH, Ganz PA (2015) Life after diagnosis and treatment of cancer in adulthood: contributions from psychosocial oncology research. Am Psychol 70(2):159–174. 10.1037/a0037875 25730722 10.1037/a0037875

[3] Eakin EG, Reeves MM, Goode AD, Winkler EAH, Vardy JL, Boyle F, Haas MR, Hiller JE, Mishra GD, Jefford M, Koczwara B, Saunders CM, Chapman K, Hing L, Boltong AG, Lane K, Baldwin P, Millar L, McKiernan S, Hayes SC (2020) Translating research into practise: outcomes from the Healthy Living after Cancer partnership project. BMC Cancer 20(1):963. 10.1186/s12885-020-07454-4 33023538 10.1186/s12885-020-07454-4 PMC7539431

[4] Escriva Boulley G, Leroy T, Bernetière C, Paquienseguy F, Desfriches-Doria O, Préau M (2018) Digital health interventions to help living with cancer: a systematic review of participants’ engagement and psychosocial effects. Psychooncology 27(12):2677–2686. 10.1002/pon.4867 30152074 10.1002/pon.4867

[5] Cuthbert CA, Farragher JF, Hemmelgarn BR, Ding Q, McKinnon GP, Cheung WY (2019) Self-management interventions for cancer survivors: a systematic review and evaluation of intervention content and theories. Psychooncology 28(11):2119–2140. 10.1002/pon.5215 31475766 10.1002/pon.5215

[6] Epplein M, Zheng Y, Zheng W, Chen Z, Gu K, Penson D, Lu W, Shu XO (2011) Quality of life after breast cancer diagnosis and survival. J Clin Oncol 29(4):406–412. 10.1200/JCO.2010.30.6951 21172892 10.1200/JCO.2010.30.6951 PMC3058286

[7] McTiernan A, Friedenreich CM, Katzmarzyk PT, Powell KE, Macko R, Buchner D, Pescatello LS, Bloodgood B, Tennant B, Vaux-Bjerke A, George SM, Troiano RP, Piercy KL, Physical Activity Guidelines Advisory C (2019) Physical activity in cancer prevention and survival: a systematic review. Med Sci Sports Exerc 51(6), 1252–1261. 10.1249/MSS.0000000000001937 10.1249/MSS.0000000000001937 PMC6527123 31095082

[8] Grimmett C, Corbett T, Brunet J, Shepherd J, Pinto BM, May CR, Foster C (2019) Systematic review and meta-analysis of maintenance of physical activity behaviour change in cancer survivors. Int J Behav Nutr Phys Act 16(1):37. 10.1186/s12966-019-0787-4 31029140 10.1186/s12966-019-0787-4 PMC6486962

[9] Tollosa DN, Holliday E, Hure A, Tavener M, James EL (2020) A 15-year follow-up study on long-term adherence to health behaviour recommendations in women diagnosed with breast cancer. Breast Cancer Res Treat 182(3):727–738. 10.1007/s10549-020-05704-4 32535764 10.1007/s10549-020-05704-4

[10] Ng HS, Roder D, Koczwara B, Vitry A (2018) Comorbidity, physical and mental health among cancer patients and survivors: an Australian population‐based study. Asia-Pac J Clin Oncol 14(2). 10.1111/ajco.12677 10.1111/ajco.12677 28371441

[11] Grant AR, Koczwara B, Morris JN, Eakin E, Short CE, Beatty L (2021) What do cancer survivors and their health care providers want from a healthy living program? Results from the first round of a co-design project. Support Care Cancer 29(8):4847–4858. 10.1007/s00520-021-06019-w 33544245 10.1007/s00520-021-06019-w

[12] Beatty L, Kemp E, Butow P, Girgis A, Schofield P, Turner J, Hulbert-Williams NJ, Levesque JV, Koczwara B (2018) A systematic review of psychotherapeutic interventions for women with metastatic breast cancer: context matters. Psychooncology 27(1):34–42 28432855 10.1002/pon.4445

[13] Santin O, Murray L, Prue G, Gavin A, Gormley G, Donnelly M (2015) Self-reported psychosocial needs and health-related quality of life of colorectal cancer survivors. Eur J Oncol Nurs 19(4):336–342. 10.1016/j.ejon.2015.01.009 25800658 10.1016/j.ejon.2015.01.009

[14] Lisy K, Langdon L, Piper A, Jefford M (2019) Identifying the most prevalent unmet needs of cancer surviviors in Australia: a systematic review. Asia Pac J Clin Oncol 15(5):e68–e78. 10.1111/ajco.13176 31215167 10.1111/ajco.13176

[15] Wang YH, Li JQ, Shi JF, Que JY, Liu JJ, Lappin J, Leung J, Ravindran A, Chen W, Qiao YL, Shi J, Lu L, Bao YP (2020) Depression and anxiety in relation to cancer incidence and mortality: a systematic review and meta-analysis of cohort studies. Mol Psychiatry 25(7):1487–1499. 10.1038/s41380-019-0595-x 31745237 10.1038/s41380-019-0595-x

[16] Jiang X, Ming WK, You JH (2019) The cost-effectiveness of digital health interventions on the management of cardiovascular diseases: systematic review. J Med Internet Res 21(6):e13166 31210136 10.2196/13166 PMC6601257

[17] Roberts AL, Fisher A, Smith L, Heinrich M, Potts HWW (2017) Digital health behaviour change interventions targeting physical activity and diet in cancer survivors: a systematic review and meta-analysis. J Cancer Surviv 11(6):704–719. 10.1007/s11764-017-0632-1 28779220 10.1007/s11764-017-0632-1 PMC5671545

[18] Leske M, Koczwara B, Blunt J, Morris J, Eakin E, Short CE, Daly A, Degner J, Beatty L (2022) Co-designing Healthy Living after Cancer Online: an online nutrition, physical activity, and psychosocial intervention for post-treatment cancer survivors. J Cancer Surviv. 10.1007/s11764-022-01284-y 36374435 10.1007/s11764-022-01284-y PMC9660094

[19] Eakin EG, Hayes SC, Haas MR, Reeves MM, Vardy JL, Boyle F, Hiller JE, Mishra GD, Goode AD, Jefford M, Koczwara B, Saunders CM, Demark-Wahnefried W, Courneya KS, Schmitz KH, Girgis A, White K, Chapman K, Boltong AG, Robson EL (2015) Healthy Living after Cancer: a dissemination and implementation study evaluating a telephone-delivered healthy lifestyle program for cancer survivors. BMC Cancer 15:992. 10.1186/s12885-015-2003-5 26690258 10.1186/s12885-015-2003-5 PMC4687340

[20] Leske M, Koczwara B, Morris J, Beatty L (2023) Evaluating the feasibility of a co-designed, online healthy living intervention for post-treatment cancer survivors: Healthy Living after Cancer Online [poster presentation]. Cancer Survivorship Conference, Adelaide, Australia.

[21] Akdemir A, Smith A, Wu VS, Rincones O, Russell H, Lyhne JD, Kemp E, David M, Bamgboje‐Ayodele A (2024) Guided versus non‐guided digital psychological interventions for cancer patients: a systematic review and meta‐analysis of engagement and efficacy. Psycho-Oncol. 10.1002/pon.6290 10.1002/pon.6290 38282223

[22] Leske M, Koczwara B, Morris J, Eakin E, Short C, Daly A, Degner J, Beatty L (2023) Feasibility and preliminary efficacy of brief coaching calls in Healthy Living after Cancer Online: a randomised control trial. International Psycho-Oncology Society World Congress, Milan, Italy. file:///C:/Users/lesk0008/AppData/Local/Downloads/ipos_2023_abstracts_booklet.1.pdf

[23] Evans HEL, Galvão DA, Forbes CC, Girard D, Vandelanotte C, Newton RU, Vincent AD, Wittert G, Kichenadasse G, Chambers S, Brook N, Short CE (2021) Acceptability and preliminary efficacy of a web- and telephone-based personalised exercise intervention for individuals with metastatic prostate cancer: the ExerciseGuide pilot randomised controlled trial. Cancers. 10.3390/cancers13235925 34885036 10.3390/cancers13235925 PMC8656540

[24] Leske M, Koczwara B, Morris J, Beatty L (2023) Modality preferences for health behaviour interventions for post-treatment cancer survivors: a theoretical investigation. Support Care Cancer. 10.1007/s00520-023-07607-8 36729337 10.1007/s00520-023-07607-8 PMC9892669

[25] Gulliver A, Calear AL, Sunderland M, Kay-Lambkin F, Farrer LM, Batterham PJ (2021) Predictors of acceptability and engagement in a self-guided online program for depression and anxiety. Internet Interv. 10.1016/j.invent.2021.100400 34026569 10.1016/j.invent.2021.100400 PMC8122006

[26] Perski O, Short CE (2021) Acceptability of digital health interventions: embracing the complexity. Transl Behav Med 11(7):1473–1480. 10.1093/tbm/ibab048 33963864 10.1093/tbm/ibab048 PMC8320880

[27] Malterud K, Siersma VD, Guassora AD (2016) Sample size in qualitative interview studies. Qual Health Res 26(13):1753–1760. 10.1177/1049732315617444 26613970 10.1177/1049732315617444

[28] Hennink M, Kaiser BN (2022) Sample sizes for saturation in qualitative research: a systematic review of empirical tests. Soc Sci Med 292:114523. 10.1016/j.socscimed.2021.114523 34785096 10.1016/j.socscimed.2021.114523

[29] Lumivero (2020) *NVivo* (Version R1) www.lumivero.com

[30] Gale NK, Heath G, Cameron E, Rashid S, Redwood S (2013) Using the framework method for the analysis of qualitative data in multi-disciplinary health research. BMC Med Res Methodol 13:117. 10.1186/1471-2288-13-117 24047204 10.1186/1471-2288-13-117 PMC3848812

[31] Bowen DJ, Kreuter M, Spring B, Cofta-Woerpel L, Linnan L, Weiner D, Bakken S, Kaplan CP, Squiers L, Fabrizio C, Fernandez M (2009) How we design feasibility studies. Am J Prev Med 36(5):452–457. 10.1016/j.amepre.2009.02.002 19362699 10.1016/j.amepre.2009.02.002 PMC2859314

[32] Braun V, Clarke V (2006) Using thematic analysis in psychology. Qual Res Psychol 3(2):77–101. 10.1191/1478088706qp063oa

[33] Braun V, Clarke V (2023) Is thematic analysis used well in health psychology? A critical review of published research, with recommendations for quality practise and reporting. Health Psychol Rev. 10.1080/17437199.2022.2161594 36656762 10.1080/17437199.2022.2161594

[34] Beatty L, Binnion C (2016) A systematic review of predictors of, and reasons for, adherence to online psychological interventions. Int J Behav Med 23(6):776–794. 10.1007/s12529-016-9556-9 26957109 10.1007/s12529-016-9556-9

[35] O’Connor S, Hanlon P, O’Donnell CA, Garcia S, Glanville J, Mair FS (2016) Understanding factors affecting patient and public engagement and recruitment to digital health interventions: a systematic review of qualitative studies. BMC Med Inform Decis Mak 16(1):120. 10.1186/s12911-016-0359-3 27630020 10.1186/s12911-016-0359-3 PMC5024516

[36] Yardley L, Spring BJ, Riper H, Morrison LG, Crane DH, Curtis K, Merchant GC, Naughton F, Blandford A (2016) Understanding and promoting effective engagement with digital behavior change interventions. Am J Prev Med 51(5):833–842. 10.1016/j.amepre.2016.06.015 27745683 10.1016/j.amepre.2016.06.015

[37] Montalescot L, Baussard L, Charbonnier E (2024) Factors associated with digital intervention engagement and adherence in patients with cancer: systematic review. J Med Internet Res 26:e52542. 10.2196/52542 39661976 10.2196/52542 PMC11669875

[38] Leung C, Leung J, Pei K, Hudec F, Shams R, Munthali D (2022) The effects of nonclinician guidance on effectiveness and process outcomes in digital mental health interventions: systematic review and meta-analysis. J of Med Internet Res 24(6). 10.2196/36004 10.2196/36004 PMC9244656 35511463

[39] McVay MA, Bennett GG, Steinberg D, Voils CI (2019) Dose–response research in digital health interventions: concepts, considerations, and challenges. Health Psychol 38(12):1168–1174. 10.1037/hea0000805 31580127 10.1037/hea0000805 PMC6861635

[40] Stan DL, Stan SM, Cutshall TF, Adams K, Ghosh MM, Clark KC, Wieneke EB, Kebede BJ, Donelan Dunlap KJ, Ruddy JK, Hazelton AM, Butts SM, Jenkins IT, Croghan BA (2020) Wellness coaching: an intervention to increase healthy behavior in breast cancer survivors. Clin J Oncol Nurs 24(3):305–315. 10.1188/20.CJON.305-315 32441691 10.1188/20.CJON.305-315 PMC7486982

[41] Dennison L, Dennison L, Morrison S, Lloyd D, Phillips B, Stuart S, Williams K, Bradbury P, Roderick E, Murray S, Michie P, Little L (2014) Does brief telephone support improve engagement with a web-based weight management intervention? Randomized controlled trial. J Med Internet Res 16(3). 10.2196/jmir.3199 10.2196/jmir.3199 PMC4004138 24681761

[42] Loughnane C, Laiti J, O’Donovan R, Dunne PJ (2025) Systematic review exploring human, AI, and hybrid health coaching in digital health interventions: trends, engagement, and lifestyle outcomes. Frontiers in Digital Health 7. 10.3389/fdgth.2025.1536416 10.3389/fdgth.2025.1536416 PMC12058678 40343215

[43] Palmer CE, Marshall E, Millgate E, Warren G, Ewbank M, Cooper E, Lawes S, Smith A, Hutchins-Joss C, Young J, Bouazzaoui M, Margoum M, Healey S, Marshall L, Mehew S, Cummins R, Tablan V, Catarino A, Welchman AE, Blackwell AD (2025) Combining artificial intelligence and human support in mental health: digital intervention with comparable effectiveness to human-delivered care. J Med Internet Res 27:e69351. 10.2196/69351 40152000 10.2196/69351 PMC12117275

[44] Mohr DC, Cuijpers P, Lehman K (2011) Supportive accountability: a model for providing human support to enhance adherence to eHealth interventions. J Med Internet Res. 10.2196/jmir.1602 21393123 10.2196/jmir.1602 PMC3221353

[45] Amireault S, Fong AJ, Sabiston CM (2018) Promoting healthy eating and physical activity behaviors: a systematic review of multiple health behavior change interventions among cancer survivors. Am J Lifestyle Med 12(3):184–199. 10.1177/1559827616661490 30202391 10.1177/1559827616661490 PMC6124968

